# Visualization of simulated small vessels on computed tomography using a model-based iterative reconstruction technique

**DOI:** 10.1016/j.dib.2017.06.024

**Published:** 2017-06-16

**Authors:** Toru Higaki, Fuminari Tatsugami, Chikako Fujioka, Hiroaki Sakane, Yuko Nakamura, Yasutaka Baba, Makoto Iida, Kazuo Awai

**Affiliations:** a Department of Diagnostic Radiology, Hiroshima University, 1–2-3 Kasumi, Minami-ku, Hiroshima 734-8551, Japan; b Department of Radiology, Hiroshima University Hospital, 1–2-3 Kasumi, Minami-ku, Hiroshima 734-8551, Japan

**Keywords:** Computed tomography (CT), CT angiography, Image reconstruction, Hybrid iterative reconstruction, Model-based iterative reconstruction

## Abstract

This article describes a quantitative evaluation of visualizing small vessels using several image reconstruction methods in computed tomography. Simulated vessels with diameters of 1–6 mm made by 3D printer was scanned using 320-row detector computed tomography (CT). Hybrid iterative reconstruction (hybrid IR) and model-based iterative reconstruction (MBIR) were performed for the image reconstruction.

**Specifications Table** *[please fill in right-hand column of the table below]*

**Table:** 

Subject area	Radiology
More specific subject area	Effect of Image reconstruction methods for small blood vessels in CT.
Type of data	Image, graph, text
How data was acquired	Phantom with simulated small vessels was scanned with CT, and it was reconstructed by hybrid IR and MBIR.
Data format	Raw, Analyzed
Experimental factors	The sharpness of the blood vessel boundary was measured with a quantitative index.
Experimental features	Radiation dose was determined by the routinely used noise level in coronary CT angiography. Adaptive Iterative Dose Reduction 3D (AIDR 3D) was used as the hybrid IR, Forward-projected model-based Iterative Reconstruction SoluTion (FIRST) was used as the MBIR.
Data source location	1-2-3 Kasumi, Minami-ku, Hiroshima, 34° 22′ 44.4′′ N; 132° 28′ 38.26′′ E
Data accessibility	The data are available with this article

**Value of the data**

• The data has described the effect of MBIR for visualizing small vessels in CT images.
• Researchers can recognize the difference in the appearance of small vessels in various reconstruction method.
• Also researchers can recognize the difference in the appearance of small vessels in various diameters.
• This data shows the superiority of MBIR for visualization of small vessels.

## 1 Experimental design, materials and methods

### 1.1 Vessel phantom

**Fig. 1:**
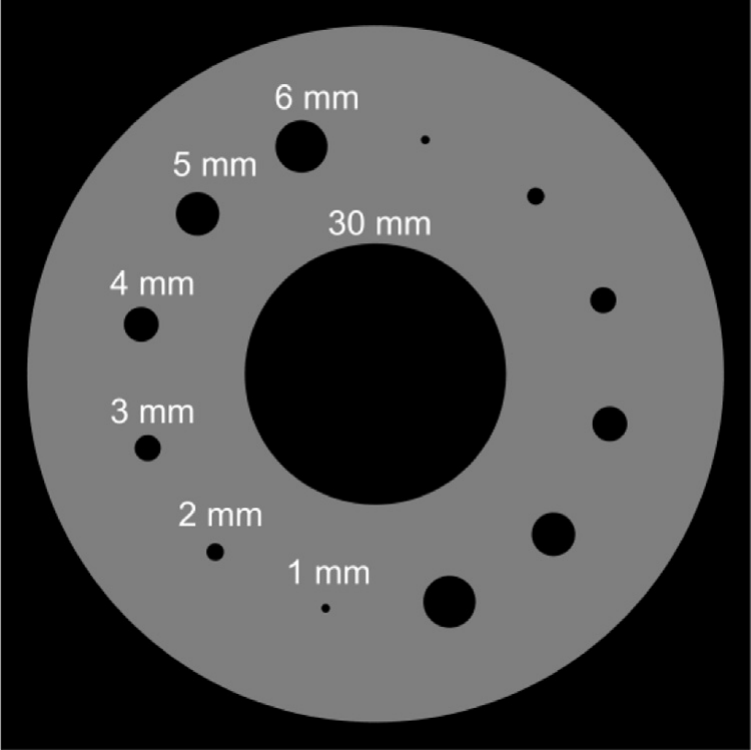
Design of our vessel phantom. The cylinders measuring 1–6 mm in diameter simulate small vessels. The 30-mm diameter cylinder in the center simulates the ascending aorta.

The vessel phantom (outer diameter 80 mm) was made by a 3D printer (Agilista 3100, KEYENCE, Osaka, Japan) and included cylinders that simulated 1-, 2-, 3-, 4-, 5-, and 6-mm vessels (Fig. 1). The aorta was simulated by a 30-mm diameter cylinder at the center of the phantom. The cylinders were filled with diluted iodine contrast material (Iohexol, Daiichi-Sankyo, Tokyo, Japan, concentration 13 mgI/ml) to simulate the vascular space.

### 1.2 CT Scanning protocol

CT scans were obtained on a 320-detector scanner (Aquilion ONE ViSION Ed., Toshiba Medical Systems Corp., Tokyo, Japan) with ECG-triggering. Scanning was at a tube voltage of 120 kV in volume-scanning mode. The tube current was set at 60 mA to emulate the routine imaging conditions (noise index 25 SD) for coronary CT angiography (CCTA). The other scanning parameters were 0.275 s rotation time, 0.5 mm slice thickness, 40 mm (0.5 mm×80 slices) z-coverage range, and 100-mm field of view.

### 1.3 CT Image reconstruction

Adaptive iterative dose reduction 3D (AIDR 3D, Toshiba Medical Systems Corp.) was used as the hybrid IR. To obtain AIDR 3D images we used three kinds of standard soft tissue convolution kernels (FC13, FC14 and FC15); they are recommended by the vendor for routine CCTA studies. Forward projected model-based iterative reconstruction solution (FIRST, Toshiba Medical Systems Corp.) with CCTA mode was used as the MBIR.

### 1.4 Data analysis and quantitative evaluation

**Fig. 2:**
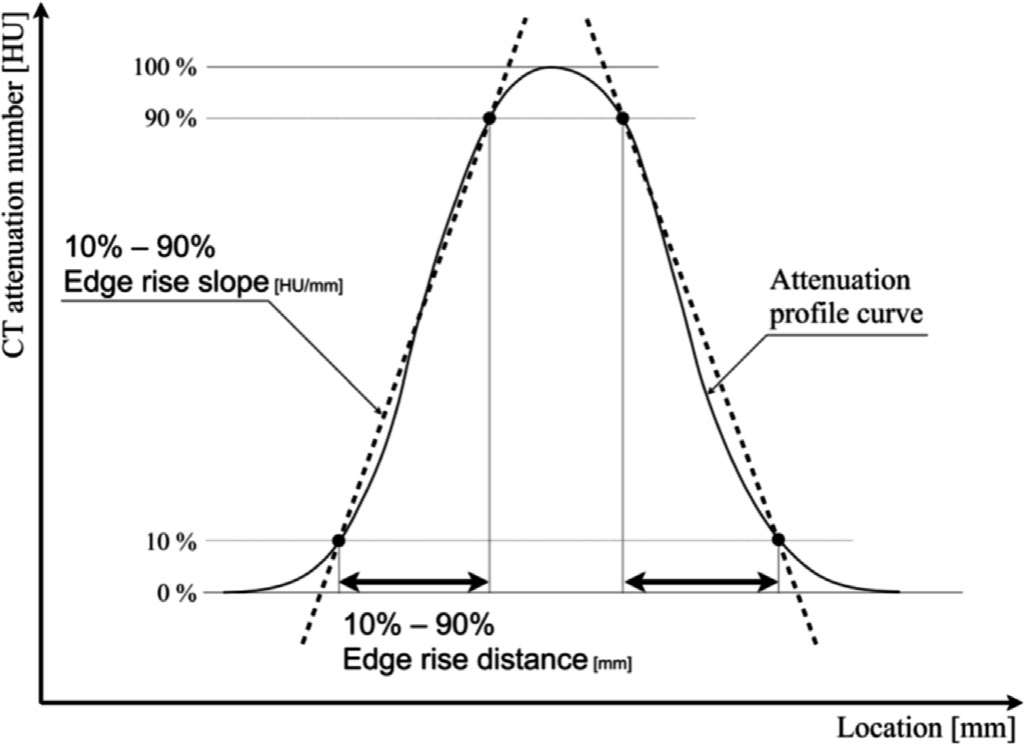
Definition of the edge rise distance (ERD) and the edge rise slope (ERS) on CT attenuation profile curves. ERD is defined as the distance between the site showing 10% and the site showing 90% of the CT number and ERS as the slope of the line connecting the 10% and the 90% HU site.

To evaluate the visibility of simulated vessels we recorded their CT attenuation profile curves (APCs) on CTA images. We used ImageJ software [1] and its particle analysis tool (Plot Profile) to generate APCs and recorded 36 APCs radially around the vessel centers at 5° intervals. As the objective edge response index on APCs we determined the 10 - 90% edge rise distance (ERD) [2] and the 10–90% edge rise slope (ERS) at the vessel boundaries (Fig. 2). The distance and slope values were examined on both sides of the simulated vascular wall, hence, a total of 72 of ERDs and 72 ERSs was obtained and they were averaged in every simulated vessel. We also recorded the peak CT attenuation number in Hounsfield units (HU, PCT-HU) on the APCs and the CT attenuation value and the standard deviation (image noise) for the simulated aorta. The signal-to-noise ratio (SNR) for each vessel was calculated by dividing PCT-HU by the image noise.

## 2 Data

**Fig. 3:**
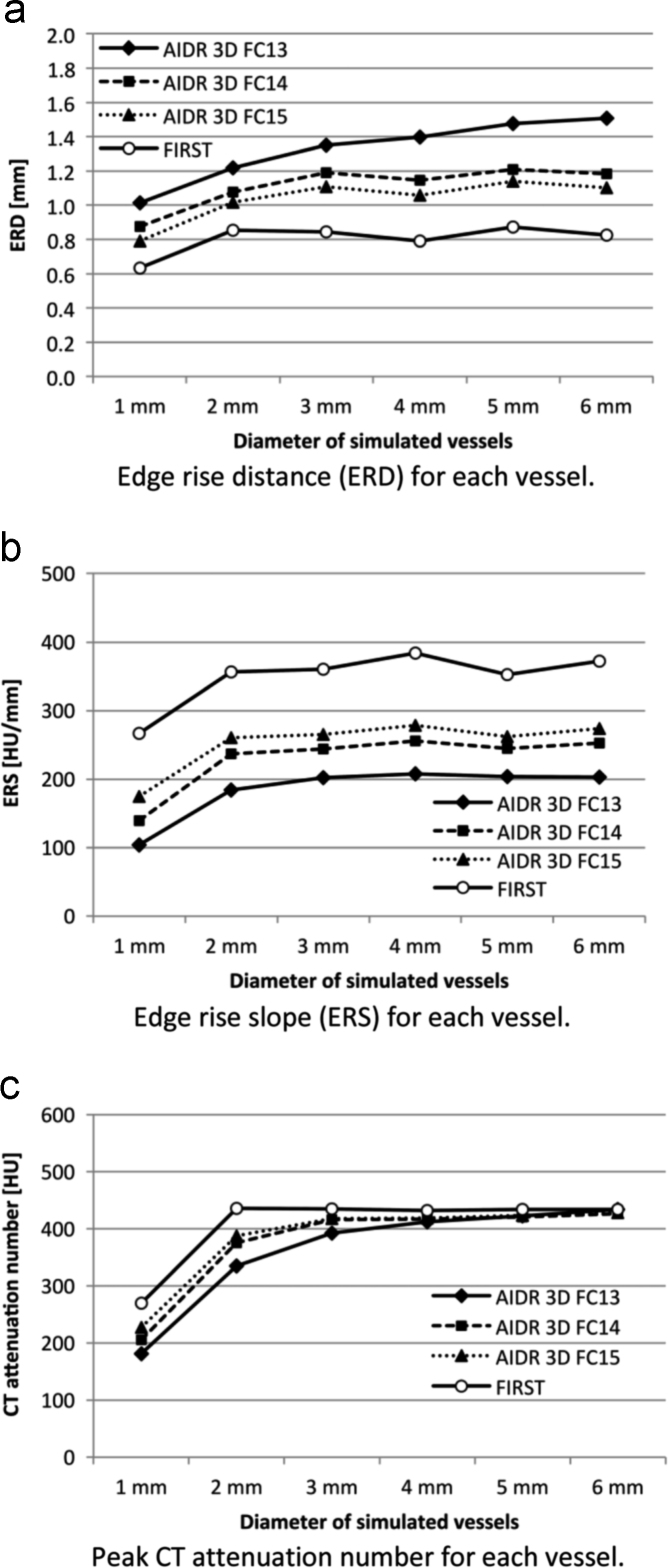
Results of attenuation profile curve analysis. On images reconstructed with FIRST, the ERD was smaller and the ERS was larger than on vessel images reconstructed with the other methods.

**Fig. 4:**
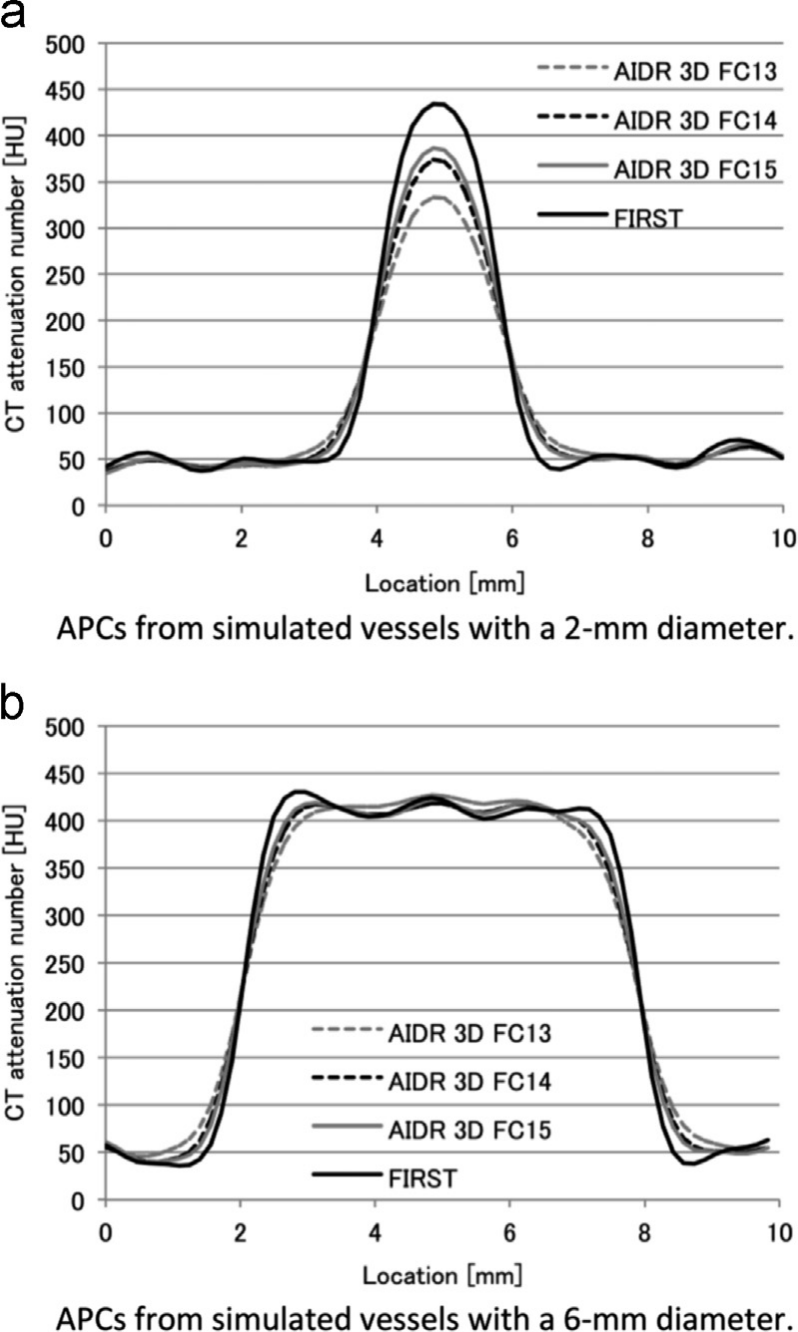
Representative attenuation profile curves (APCs). The peak CT number of 2-mm vessels was strongly affected by the applied reconstruction algorithm; with FIRST we recorded the highest HU value. The HU value for 2-mm vessels was similar with all reconstruction algorithms we investigated.

**Fig. 5:**
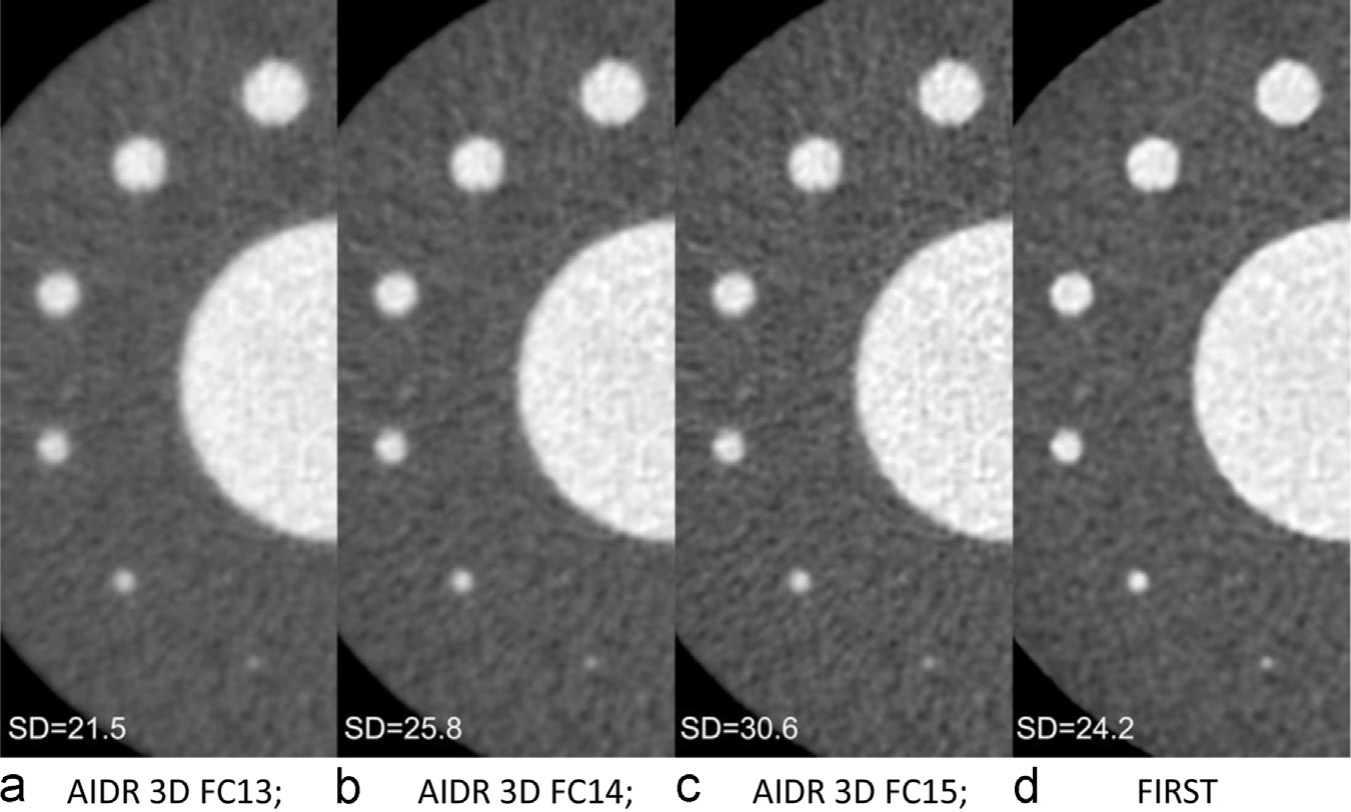
Representative images of our vessel phantom. The effect of the convolution kernels changed gradually from FC13 to FC15. With FC13 the image noise and image sharpness were the lowest; with FC15 they were the highest. The image noise on FIRST- and AIDR 3D-FC14 images was comparable.

The ERDs and ERSs measured on vessels with different diameters are shown in Figs. 3a and 3b. With FIRST the ERD was smaller and the ERS was larger than with AIDR 3D. The PCT-HU value of the vessels is shown in Fig. 3c. For 6-mm vessels the PCT-HU obtained with the different reconstruction methods was similar. On images reconstructed with AIDR 3D and any of the convolution kernels-, the smaller the vessel diameter, the lower was the PCT-HU value. On FIRST images, the PCT-HU value were constantly stable and highest at the vessel diameter of 2–5 mm. The aortic PCT-HU of images acquired with AIDR 3D-FC13, AIDR 3D-FC14, and AIDR 3D-FC15 and with FIRST was 412.3, 410.6, 410.8, and 411.1, respectively. Representative APCs are shown in Fig. 4. The image noise on those images was 21.5, 25.8, 30.6, and 24.2, respectively (Fig. 5); it was comparable on AIDR 3D-FC14- and FIRST images (24.2 and 25.8 HU, respectively).

**Fig. 6:**
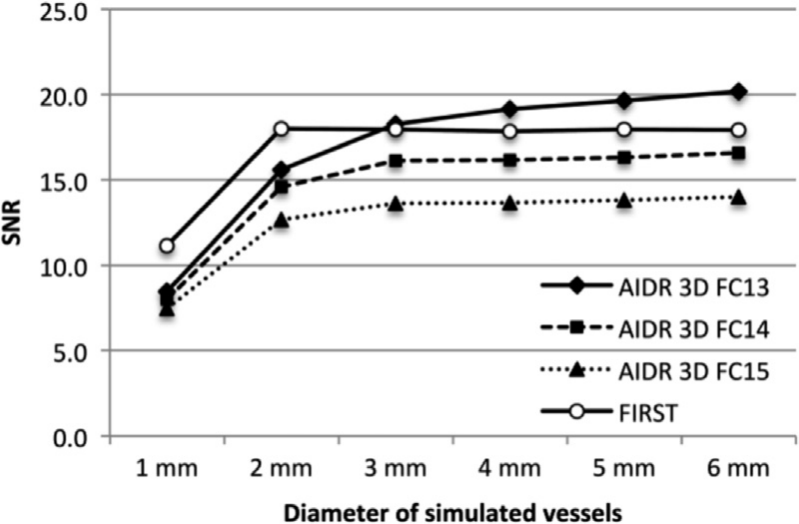
Signal-to-noise ratios (SNRs). The SNR of simulated vessels with a diameter of up to 3 mm was higher on FIRST- than on AIDR 3D-FC14 and AIDR 3D-FC15 images. For vessels with a diameter greater than 4 mm the SNR value was higher on AIDR 3D-FC13- than FIRST images.

As shown in Fig. 6, the SNR on AIDR 3D-FC14 and AIDR 3D-FC15 images of vessels was lower than on FIRST scans for vessels of all diameters examined. We obtained higher SNR values for vessels whose diameter exceeded 4 mm when AIDR 3D-FC13 rather than FIRST was applied.
