# Supplementary material for: Visualization of simulated small vessels on computed tomography using a model-based iterative reconstruction technique
Source: Data Brief. 2017 Jun 16;13:437–43. doi: 10.1016/j.dib.2017.06.024 (PMC5484979; doi:10.1016/j.dib.2017.06.024)
Supplement: Supplementary file 1 — Supplementary material [file mmc1.docx]

**Visualization of simulated small vessels on computed tomography using a model-based iterative reconstruction technique**

Toru Higaki, Fuminari Tatsugami, Chikako Fujioka, Hiroaki Sakane, Yuko Nakamura, Yasutaka Baba, Makoto Iida, Kazuo Awai.

**COI Information**

Toru Higaki:

N/A.

Fuminari Tatsugami:

N/A.

Chikako Fujioka:

N/A.

Hiroaki Sakane:

N/A.

Yuko Nakamura:

N/A.

Yasutaka Baba:

N/A.

Makoto Iida:

N/A.

Kazuo Awai:

Research Grant,Toshiba Medical Systems, Ongoing, Paid to the institution

Research Grant, Hitachi, Ongoing, Paid to the institution

Research Grant, Fujitsu, Ongoing, Paid to the institution

Research Grant, Bayer Yakuhin, Ongoing, Paid to the institution

Research Grant, Daiichi Sankyo, Ongoing, Paid to the institution

Research Grant, Eizai, Ongoing, Paid to the institution

Research Grant, Fuji Yakuhin, Ongoing, Paid to the institution
